# Incorporating Volunteering Into Treatment for Depression Among Adolescents: Developmental and Clinical Considerations

**DOI:** 10.3389/fpsyg.2021.642910

**Published:** 2021-05-05

**Authors:** Parissa J. Ballard, Stephanie S. Daniel, Grace Anderson, Linda Nicolotti, Elimarie Caballero Quinones, Min Lee, Aubry N. Koehler

**Affiliations:** ^1^Department of Family and Community Medicine, Wake Forest School of Medicine, Winston-Salem, NC, United States; ^2^Department of Psychology, Wake Forest University, Winston-Salem, NC, United States; ^3^Department of Pediatrics, Wake Forest School of Medicine, Winston-Salem, NC, United States; ^4^Department of Psychiatry, Wake Forest Baptist Medical Center, Winston-Salem, NC, United States

**Keywords:** depression, adolescents, volunteering, mental health, treatment, affective disorders

## Abstract

Volunteering, or taking part in unpaid work for the benefit of others, can be a powerful positive experience with returns to both individual well-being and community projects. Volunteering is positively associated with mental health in observational studies with community samples but has not been systematically examined as a potential part of treatment interventions with clinical adolescent samples. In this manuscript, we review the empirical evidence base connecting volunteerism to mental health and well-being, outline potential mechanisms based in the theoretical literature from developmental science, and discuss the existing clinical approaches that support community volunteering as a part of treatment. Drawing on this review, we propose that including volunteering as a component of clinical treatment approaches for adolescent depression can be a powerful intervention for adolescents.

## Introduction

Affective disorders, such as depression, increase during adolescence (Beesdo et al., 2009) and initial episodes peak between the ages of 14 and 24 (Kessler et al., 2001) influencing well-being over the life course (Lewandowski et al., 2013). Many effective pharmacological and non-pharmacological treatment approaches exist for depression (Butler et al., 2006; Beck, 2011; Weersing et al., 2017). Community volunteerism may be one underutilized, or at least under-researched, strategy to use in conjunction with clinical treatment for adolescent depression. Below, we review the empirical evidence connecting volunteerism to mental health, outline potential mechanisms based in developmental science, and discuss the existing clinical approaches that support community volunteering as a part of treatment. We propose that it is worth examining the feasibility and utility of including volunteering (also referred to as volunteerism or community volunteering) within clinical approaches to treating adolescent depression.

## Community Volunteering and Mental Health

Volunteering, or taking part in unpaid work for the benefit of others, can be a powerful positive experience with returns to both individual well-being and community projects. Volunteering is robustly positively associated with well-being (Thoits and Hewitt, 2001) and mental health according to cross-sectional and longitudinal observation studies (see Jenkinson et al., 2013 for a review). The empirical evidence available, however, is heavily focused on older adults (Lum and Lightfoot, 2005; Anderson et al., 2014). Although less studied, rigorous longitudinal studies have also revealed positive associations between volunteering and mental health over time in community samples of adolescents and young adults (Kim and Morgül, 2017; Wray-Lake et al., 2017; Ballard et al., 2019). Despite considerable *observational* evidence with community samples, there has been less research on volunteering as an *intervention* to promote health and well-being. Jenkinson et al. (2013) found that longitudinal studies support favorable associations between volunteering and depression, life satisfaction, and well-being; however, the smaller set of experimental studies available – mostly with older adult community samples – do not confirm these associations. Notably, the one study using an RCT design with a school-based sample of adolescents found that volunteering benefited adolescents’ physical health (Schreier et al., 2013), but did not measure mental health outcomes. While the observational findings with community samples are promising, there has been less focus on adolescents and young adults, the mechanisms explaining the link between volunteering and depression are largely untested, and the role of volunteering as an intervention for *treating* depression in clinical samples is underexplored.

## Conceptualizing Mechanisms Explaining Why Volunteering Might Promote Positive Mental Health Among Adolescents

Many theories and frameworks from sociology, psychology, and public health propose how volunteerism might benefit mental health. For example, identity-based theories, social structural and social network theories, and motivational theories have been applied to explain why volunteering might function to promote positive mental and psychological well-being (see Piliavin and Siegl, 2015 for a detailed discussion). Several reviews and chapters summarize theory and findings and important considerations for understanding the potential associations between volunteering and mental health (e.g., Konrath and Brown, 2013; Piliavin and Siegl, 2015; Creaven et al., 2018). To date, research has focused on community samples and not clinical samples, observational and not intervention designs, and has characterized associations between naturally occurring volunteering and mental health and not necessarily conceptualize volunteering as a potential part of clinical treatment approaches. The present manuscript summarizes and applies ideas from developmental science, in particular, as we propose volunteering as a potentially powerful part of clinical treatment for adolescent mood disorders such as depression.

### Developmental Focus on Adolescence

From a developmental perspective, there are many reasons to focus on the role of volunteerism in mitigating depressive symptoms among adolescents. First, volunteering can provide a positive and meaningful social role for adolescents. Adolescents have the developmentally salient task of carving out meaningful social roles in society often in contexts that offer them few opportunities to do so (Eccles et al., 1993; Yeager et al., 2017; Fuligni, 2019). Second, recent discoveries in developmental science and neuroscience point to early adolescence as a specific window of opportunity during which youth are especially sensitive to social and affective influences (Crone and Dahl, 2012; Telzer, 2016); therefore, positive experiences that are socially-based and affectively positive might have a powerful sway in adolescent decision-making and health trajectories. This type of experience might be foundational for “positive spirals” in adolescence proposed to lead to long-term positive mental health (Crone and Dahl, 2012). Third, the talents and skills of adolescents are an asset to and often untapped resource within our communities. Many scholars propose that including youth in community-based projects not only promotes developmental outcomes for youth, but also benefits the projects that youth engage in through research, policy, or solving community problems (Ballard and Syme, 2015; Ozer, 2017).

### Potential Mechanisms

**Figure 1:**
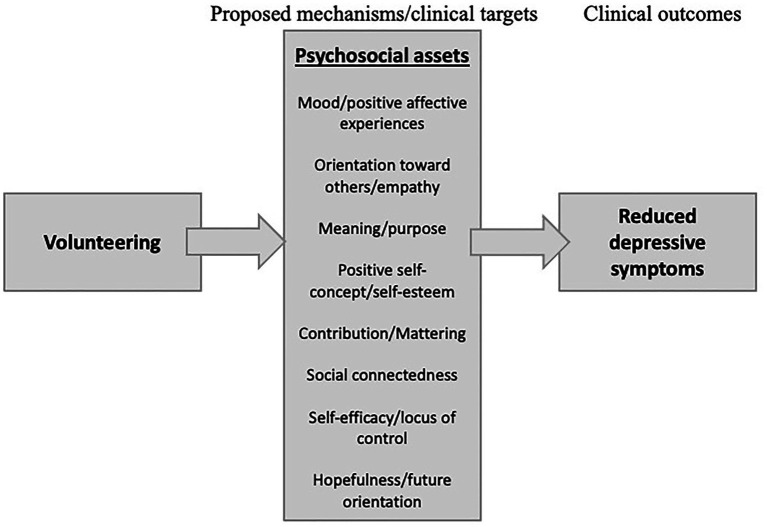
Prosposed mechanisms/clinical targets whereby volunteering affects depressive symptoms.

Volunteering can provide the opportunity to build many psychosocial assets with developmental and clinical significance for adolescents with depression. These psychosocial assets reviewed below (Figure 1) are targets for many clinical therapies aimed at treating mood disorders such as depression.

#### Mood/Positive Affective Experiences

One of the most straightforward mechanisms that might be at play is that it feels good to help others (Brown et al., 2003; Piliavin and Siegl, 2007; Poulin et al., 2013; Inagaki and Orehek, 2017). Helping and supporting others can lift one’s mood, and sustained opportunities to do so might benefit adolescents with depression both in the short-term and the long-term.

#### Orientation Toward Others/Empathy

One prominent idea is that volunteering facilitates an orientation toward others and decreases self-focus (Oman et al., 1999; Malin et al., 2015). In short, volunteering might help people focus on themselves less and on others more and might help teens develop perspective that allows them to deal with stress (e.g., Raposa et al., 2016) by situating or re-framing the normative stressors they face. This might be especially useful in adolescent depression where extreme self-focus can lead or contribute to rumination on one’s own problems; volunteering might be one way to reduce self-focus that can be associated with depression.

#### Meaning/Purpose

A developmental task of adolescence is carving out one’s identity and purpose in relation to the world around them (Erikson, 1968; Damon, 2008). Volunteering can offer a venue to develop and enact a sense of purpose. This may be formative for adolescents whose depression is characterized by questions about self-worth and lack of purpose.

#### Positive Self-Concept/Self-Esteem

Holding a generally positive view of oneself, operationalized as a positive self-concept or gain self-esteem, is an important component of mental health. Community volunteering can provide meaningful social role opportunities that offer teens a chance to build skills and feel competent (Moore and Allen, 1996; Ryan and Deci, 2000) which may shape positive self-concepts, especially benefiting young people with depression (Beck, 1967).

#### Contribution/Mattering

Adolescents have a need to contribute to the world around them (Fuligni, 2019). Volunteering is one venue to contribute, thus potentially increasing a sense that one matters and has a meaningful role to play (Keyes, 1998; Elliott et al., 2005; Piliavin and Siegl, 2007). Although contribution may be a basic need for all adolescents, it may also fill a unique need for adolescents whose depression obscures opportunities and accomplishments in the realm of contributing to others.

#### Social Connectedness

Volunteering often involves opportunities to connect with others in one’s community. Such social connectedness has been shown to be critical in explaining links between volunteering and depression (Creaven et al., 2018).

#### Self-Efficacy/Locus of Control

Adolescence is a life stage characterized by increasing desire for autonomy and relative lack of control opportunities (Eccles et al., 1993). Empowerment theory and programming point out the importance of teens having the chance to develop self-efficacy and exert control (Zimmerman, 1995; Ozer, 2017), all the more important perhaps for teens with depression.

#### Hopefulness/Future Orientation

Adolescence is a time of looking toward the future as young people define their identity and consider their future goals, aspirations, and opportunities (Nurmi, 1991, 2005). Depression is often characterized by a sense of hopelessness and negative views about the future (Beck, 1967), which may impede teens’ ability and desire to focus on their “future possible selves” (Oyserman et al., 2004). Volunteering might expose young people to possibilities for their futures and foster a sense of hope as they develop skills that might help them define future roles for themselves.

## Clinical Settings: Incorporating Volunteering Into Treatment for Depression Among Adolescents

Importantly, the observational evidence linking volunteering with better mental health in adolescence has been conducted with community samples. We suggest that volunteering might be effectively applied as part of comprehensive approaches to treating affective disorders among adolescents receiving clinical treatment. Volunteering fits within the conceptual basis for existing evidenced-based approaches to treating adolescent depression. In particular, we believe that volunteerism finds resonance with the underlying ideas of cognitive-behavioral and behavioral approaches and positive psychology interventions (Reinecke et al., 1998; Butler et al., 2006; David-Ferdon and Kaslow, 2008).

### Cognitive-Behavioral Approaches

In Beck’s foundational cognitive-behavioral therapy (CBT) approach to treatment, he discusses the “cognitive triad,” which is the idea that people with depression tend to hold a set of negative beliefs about themselves, the world, and their future (Beck, 1967). CBT uses cognitive techniques to target distorted and negative thinking about the self, world, and the future. Volunteering potentially would allow teens to interrupt negative thoughts about the self and develop positive ones by building self-efficacy and positive self-regard. Volunteering could shape cognitions about the world by helping teens form positive connections to, and perceptions of, others. And volunteering might affect how teens think about their futures *via* developing a sense of hopefulness, meaning, and purpose. Thus, volunteering fits conceptually within a CBT framework.

### Behavioral Approaches

Behavioral activation (BA) is a treatment approach that focuses in part on “increasing engagement in adaptive activities (which often are those associated with the experience of pleasure or mastery; Dimidjian et al., 2011, p. 3–4).” Importantly, BA is tailored to the unique needs, patterns, and goals of clients and can be individualized and implemented with flexibility and creativity (Dimidjian et al., 2014). If used by clinical providers, volunteerism is likely to be used as one of the many potential modalities for BA. Activity scheduling is another efficacious behavioral approach to treating depression that includes learning the connections between one’s daily activities and moods and aiming to increase pleasant activities and positive interactions with one’s environments (Cuijpers et al., 2007). Volunteerism may be an activity that some clinicians recommend within this approach, but could be incorporated more explicitly within activity scheduling treatments. Thus, there may be a clinical precedent and rationale for suggesting volunteerism within treatment, but there is not a systematic approach for doing so or a clinical evidence base to guide the implementation of such an approach.

### Positive Psychology Interventions

Volunteerism as a component of treatment for adolescent depression also fits conceptually with clinical intervention approaches that focus on positive psychology. These interventions differ somewhat from existing approaches targeting negative affective systems in treating depression (Sin and Lyubomirsky, 2009). Examples of positive psychology interventions include those that target positive affective systems (Taylor et al., 2017) and those focus on building psychological capital (Song et al., 2019). The Positive Activity Intervention (PAI) is a 10-session intervention delivered by providers which targets positive emotions, cognitions, and behaviors. Empirically, PAI increased positive affect and psychological well-being pre- to post-treatment and decreased negative affect and symptoms of depression (Taylor et al., 2017). The Psychological Capital Intervention (PCI; Song et al., 2019) is a 4-session intervention delivered by providers which targets hope, optimism, self-efficacy, and resilience. Similarly to the PAI, an empirical study of the PCI found that the PCI intervention increased psychological capital and reduced depression symptoms from pre- to post-treatment compared to a control group (Song et al., 2019). Volunteering similarly has the potential to build positive emotions, cognitions, and skills.

## Treatment Intervention Concept: Considerations and Challenges

Volunteering is an innovative strategy in treating adolescent depression that fits within existing clinical treatment approaches; however, there are many open questions with regard to who might benefit from volunteering, how it might operate for adolescent development, and how to implement it in clinical settings. Below, we raise conceptual questions and consider challenges to implementing volunteering into treatment in clinical settings.

First, is volunteering “better” than other meaningful activities? Although volunteering offers some specific benefits compared to other activities – such as the chance to make a meaningful contribution to others – activities such as sports or theater offer similar opportunities for mastery experiences and building confidence. Future research can specify whether and which mechanisms are targeted by volunteering and which are common across activities.

Relatedly, not all volunteering opportunities offer equally high quality experiences. Issues of personal choice and fit are critical to teens receiving benefits of community volunteering. In a clinical setting, teens may be best served by having a menu of high-quality local options to choose from so that they can pursue their own interests. This is important to ensure that teens know about local organizations that accept young volunteers given that some have age requirements. Further, a menu of options should list organizations that view teens as resources to the community and provide enough structure and support to create meaningful experiences for young people. In particular, organizations that have a track record of working with teens to co-design volunteer opportunities can ensure a meaningful and positive developmental context for young people where they are viewed as valuable assets and have the chance to develop a positive view of themselves. For example, teens who love animals may derive meaning from working at an animal rescue center, whereas teens who love reading may find a good fit with volunteering in a library setting. This is especially important since volunteering may be most beneficial when intrinsically motivated (Konrath et al., 2012) which may be only partially the case if suggested in a clinical treatment setting. Volunteering can take many forms and can be social or solo; this is also a matter of fit and preference. Future studies can examine which forms and dimensions of volunteer experiences, if any, are universally beneficial and which are more a matter of person-opportunity fit.

Second, what “dose” would be necessary to have salutary effects on mechanisms such as meaning, hope, and self-efficacy? Correlational evidence has pointed to potential answers such as 2–3 h per week (Van Willigen, 2000; Morrow-Howell et al., 2003) and 1–10 h per month (Choi and Kim, 2011). Volunteering can strain adolescents’ already busy schedules; therefore, future studies with adolescents specifically will help clarify the dose/threshold questions to understand what amount of volunteering is clinically meaningful without adding time stressors.

Third, volunteering in one’s community presents logistical challenges with implications for equity. A major barrier to participating in many volunteer efforts is that transportation is required. For younger teens, this means reliance on parents’ availability and willingness to transport teens. Parents must then have access to transportation, have time to devote to this outside of work and other responsibilities, and must see value in supporting teens’ volunteering. For older adolescents, this means access to personal transportation, such as a car to use or reliable public transportation and having time available to spend in unpaid activities. These barriers result in a major challenge to equity; teens with transportation access and recreational time to spend in unpaid activities tend to be from higher socioeconomic backgrounds, thus potentially leaving behind teens who may benefit from volunteering but who do not have the resources to allow them to volunteer. Future research and practical efforts must apply creativity to problem-solving, for example, seeking out meaningful opportunities available within teens’ schools or accessible community organizations and/or providing transportation vouchers, to meet these challenges. The global pandemic of COVID-19 has highlighted the challenge of in-person volunteerism in the context of safety concerns and has also presented opportunities to think creatively about virtual volunteerism efforts that might benefit young people and solve transportation barriers. Research would need to attend to the potential benefits and limitations to volunteering in a virtual way, as such efforts lose some potentially important opportunity for personal connection.

Fourth, motivational challenges and anhedonia that are characteristic of affective disorders such as depression might make it hard for adolescents to begin volunteering. It might be hard for adolescents struggling with depression to see what activities would be meaningful and to take initiative and follow through with pursuing volunteering. Research must investigate whether adolescents with mild/moderate depression would want to participate in volunteering and, if so, whether it would be feasible for them to follow through with volunteering given the motivational and logistical challenges.

Fifth, who would benefit most from volunteering? While positive and meaningful volunteering experiences have the potential to benefit many teens, many issues are unresolved regarding which teens would benefit most and at what point in their treatment. Issues such as clinical severity and time course of treatment will be important for clinical research and practice to grapple with. In addition, typical clinical approaches rely on interventions delivered by providers during clinical interactions. Clinicians may encourage patients to choose activities, such as volunteering, and give “homework” structured around specific goals and problem-solving about barriers that they subsequently discuss in treatment sessions. Reflecting on one’s experience is an important part of volunteering experiences (Van Goethem et al., 2014) and clinicians can provide opportunities for this. However, clinicians may not be able to provide an intensive infrastructure to support patients in finding a meaningful volunteer opportunity; this may require additional resources.

Sixth, while this manuscript specifically focuses on the potential role of volunteering as a supplement to clinical treatment approaches, it is also worth considering the potential of volunteering more broadly in the continuum of care and in clinical settings other than one-on-one treatment. Volunteering might also play a role in preventive interventions to support adolescents’ positive mental health, such as those delivered in school or community settings rather than in clinical offices. This would extend empirical research documenting when and how volunteering supports students’ academic and social outcomes (e.g., Moore and Allen, 1996). Schools that focus on the development of the “whole child” might prioritize offering meaningful opportunities for students to engage positive in their communities, thus providing an opportunity for intersectoral cooperation between health and education systems. Group treatment approaches can also incorporate volunteering and may provide added benefits of social settings to reflect on the meaning of volunteering for one’s life.

Finally, potential downsides of volunteering must also be considered. Not all volunteering experiences are positive. It is important that the volunteer activity be well-structured and supportive. Volunteer experiences where teens do not feel respected or useful might harm their feelings of efficacy and confidence rather than bolster them. Volunteering can also be stressful, can become a time burden or an unwelcome obligation, and can expose teens to issues that would benefit from debriefing with understanding adults. Although such downsides are important to consider, when volunteering is happening against the backdrop of additional clinical treatment, clinical providers can help teens navigate these issues. Volunteerism should not be considered by itself as treatment for depression; the comprehensive approach suggested – adding volunteering alongside treatment as usual – offers a way to mitigate downsides in a safe setting.

## Conclusion

It remains an empirical question what the role is of volunteering in preventing and treating affective disorders such as depression. Community volunteering holds promise as a cost-effective and scalable part of treatment for adolescent depression that can also strengthen communities; it is worth examining whether and how to incorporate volunteering within clinical treatment approaches. Future studies are needed to test the feasibility and utility of clinical providers using community volunteerism, embedded within their existing treatment plans, with teens dealing with depression.

## Data Availability Statement

The original contributions presented in the study are included in the article/supplementary material, further inquiries can be directed to the corresponding author.

## Author Contributions

PB conceptualized and led the writing for this manuscript. SD contributed to the conceptualization and writing. GA contributed to the literature review. SD, GA, AK, LN, EC, and ML contributed to discussing, framing, reviewing the literature, and editing the manuscript. All authors contributed to the article and approved the submitted version.

### Conflict of Interest

The authors declare that the research was conducted in the absence of any commercial or financial relationships that could be construed as a potential conflict of interest.
